# Eliciting Preferences for the Uptake of Smoking Cessation Apps: Discrete Choice Experiment

**DOI:** 10.2196/37083

**Published:** 2025-01-14

**Authors:** Dorothy Szinay, Rory A Cameron, Andy Jones, Jennifer A Whitty, Tim Chadborn, Jamie Brown, Felix Naughton

**Affiliations:** 1 Behavioural and Implementation Science Group School of Health Sciences University of East Anglia Norwich United Kingdom; 2 Addiction Research Group Faculty of Medicine and Health Sciences University of East Anglia Norwich United Kingdom; 3 Behavioural Science and Health University College London London United Kingdom; 4 Norwich Medical School University of East Anglia Norwich United Kingdom; 5 Applied Research Collaboration East of England National Institute for Health Research Cambridge United Kingdom; 6 The Centre for Research in Public Health and Community Care University of Hertfordshire Hatfield United Kingdom; 7 Evidera London United Kingdom; 8 Behavioural and Social Sciences Team Department of Health and Social Care London United Kingdom; 9 SPECTRUM Consortium London United Kingdom

**Keywords:** discrete choice experiment, uptake, engagement, mHealth, smartphone app, smoking cessation, health app, behavior change, TDF, theoretical domains framework, mobile phone

## Abstract

**Background:**

If the most evidence-based and effective smoking cessation apps are not selected by smokers wanting to quit, their potential to support cessation is limited.

**Objective:**

This study sought to determine the attributes that influence smoking cessation app uptake and understand their relative importance to support future efforts to present evidence-based apps more effectively to maximize uptake.

**Methods:**

Adult smokers from the United Kingdom were invited to participate in a discrete choice experiment. Participants made 12 choices between two hypothetical smoking cessation app alternatives, with five predefined attributes reflecting domains from the theoretical domains framework: (1) monthly price of the app (environmental resources), (2) credible source as app developer (social influence), (3) social proof as star rating (social influence), (4) app description type (beliefs about consequences), and (5) images shown (beliefs about consequences); or opting out (choosing neither app). Preferences and the relative importance of attributes were estimated using mixed logit modeling. Willingness to pay and predicted uptake of the most and least preferred app were also calculated.

**Results:**

A total of 337 adult smokers completed the survey (n=168, 49.8% female; mean age 35, SD 11 years). Participants selected a smoking cessation app rather than opting out for 90% of the choices. Relative to other attributes, a 4.8-star user rating, representing social proof, was the strongest driver of app selection (mean preference parameter 2.27, SD 1.55; 95% CI 1.95-2.59). Participants preferred an app developed by health care–orientated trusted organization (credible source) over a hypothetical company (mean preference parameter 0.93, SD 1.23; 95% CI 0.72-1.15), with a logo and screenshots over logo only (mean preference parameter 0.39, SD 0.96; 95% CI 0.19-0.59), and with a lower monthly cost (mean preference parameter –0.38, SD 0.33; 95% CI –0.44 to –0.32). App description did not influence preferences. The uptake estimate for the best hypothetical app was 93% and for the worst, 3%. Participants were willing to pay a single payment of up to an additional US $6.96 (UK £5.49) for 4.8-star ratings, US $3.58 (UK £2.82) for 4-star ratings, and US $2.61(UK £2.06) for an app developed by a trusted organization.

**Conclusions:**

On average, social proof appeared to be the most influential factor in app uptake, followed by credible source, one perceived as most likely to provide evidence-based apps. These attributes may support the selection of evidence-based apps.

## Introduction

Smoking is one of the leading risk factors for noncommunicable diseases worldwide [1]. Supporting people to quit smoking is a primary concern for public health [2]. One approach is the use of apps, which can be effective for smoking cessation [3]. Many are available on commercial app stores but low uptake and suboptimal engagement with effective health apps is common [4,5]. Commercial app stores generally omit app quality measures and provide insufficient information about apps [6].

Curated health app portals are websites that present a list of selected health apps and can provide access to high-quality smoking cessation apps developed by trusted organizations [7]. This could increase the uptake of effective smoking cessation apps among smokers and decrease the risk that apps are installed primarily due to popularity, as opposed to potential effectiveness, from commercial app stores [8]. In some countries, health organizations considered as being trusted organizations, such as the Office for Health Improvement and Disparities and the National Health Service (NHS) in the United Kingdom, offer such portals (Better Health, NHS Apps Library), or the Digital Health Applications (DiGA) directory in Germany [7].

There is extensive literature on engagement with health apps [9-14], but the evidence about factors influencing their uptake is limited. We have identified several factors that appear to influence the uptake and engagement of these apps and explored views on curated health app portals, primarily the NHS “NHS Apps Library,” which since then was decommissioned, and the Office for Health Improvement and Disparities Better Health (formerly known as Public Health England’s “One You” website) [7,10]. We found a common discrepancy between user needs and what an app offers, such as the perceived utility of the app, which refers to the way apps are presented, including the images shown and the description of the apps [7,10,15]. App users have also expressed disappointment with the presentation of apps on app portals [7].

The uptake of health apps may also be primarily affected by social influences such as social proof represented by ratings of an app [7,16,17]. However, highly rated apps do not necessarily mean evidence-based content and functionality [6]. Although highly rated smoking cessation apps appear better tailored to individual needs [17], other evidence suggests that there is only a weak association between the quality of a smoking cessation app and its popularity [16,18].

There is limited evidence on which factors are likely to drive the uptake of apps and no studies investigating smoking cessation app uptake from a curated portal. This paper aims to determine preferences for the uptake of a smoking cessation app by applying a discrete choice experiment (DCE) survey. The findings may provide preliminary insights to help health app portal developers present smoking cessation apps in ways that align with user preferences, potentially supporting the selection of evidence-based apps on curated health platforms. The survey further assessed a series of factors influencing the uptake of and engagement with smoking cessation apps to better understand to what extent these factors are facilitators or barriers.

## Methods

### DCE Development

DCE is a methodology premised on individuals choosing the option that offers them the greatest utility, and assumes that utility is a function of the importance an individual assigns to characteristics associated with a product [19]. This DCE provides a series of choices of two alternatives of a product or service (here referred to as App 1 and App 2), known as choice tasks. Each alternative app is described by a set of predefined attributes, with 2 or more levels.

The development of this DCE, described in detail elsewhere [20], followed best practice guidance [21-23]. The development was further informed by discussion with stakeholders, including patient and public involvement representatives, and involved the following steps: (1) identification of attributes and attribute levels, (2) development of the experimental design, (3) piloting and survey amendment, (4) data collection, and (5) data analysis.

This paper adheres to the principles outlined in the DIRECT (Discrete Choice Experiment Reporting) checklist [24] to ensure comprehensive and transparent reporting of findings (see Multimedia Appendix 1 for the checklist).

### Attributes and Levels

A systematic review [10], and interview and think-aloud study [7] informed the selection of relevant factors mapped under the theoretical domains framework (TDF) that influence the uptake of health apps, hereby attributes of this study. The TDF informed our previous research as it provides comprehensive coverage of theory-driven domains of behavior [25].

The research team assessed the relevance and feasibility of the attributes identified in the previous stages and narrowed down the selection of potential attributes. The selection of attributes is described elsewhere [20]. The selected attributes and their levels are shown in Table 1. For the “who developed the app” attribute, we used “NHS Digital,” which is a widely trusted organization in the United Kingdom, and “Mhealth Essentials Ltd” as a hypothetical company.

**Table 1:** The attributes and attribute levels included in the DCE^a^ conducted between December 2020 and February 2021 to explore preferences for smoking cessation apps among adult smokers in the United Kingdom.

TDF^b^ construct	Attributes	Attribute levels
Environmental resources (cost)	The monthly price of the app	US $0 (UK £0) US $3.79 (UK £2.99) US $7.60 (UK £5.99) US $11.40 (UK £8.99)
Social influence (credible source)	Who developed the app	Does not say “Mhealth Essentials Ltd” “NHS^c^ Digital”
Social influence (social proof)	The ratings of the app	Does not show 3.2 stars 4 stars 4.8 stars
Beliefs in consequences (perceived utility of the app)	App description	Generic, to create a rough idea of what the app is about without getting into details of app features Short with some details about app features Long and detailed description of the app and its features
Beliefs in consequences (perceived utility of the app)	Images	Shows the logo of the app Shows the screenshot of the app Shows the logo and screenshot of the app

^a^DCE: discrete choice experiment.

^b^TDF: theoretical domains framework.

^c^NHS: National Health Service.

### Experimental Design

Participant preferences were estimated using conditional logit regression to model their choices. While a mixed logit model was used in the final analysis, the design assumed the use of McFadden’s conditional logit model: this is the most widely used approach in choice modeling [26,27], and it provides a starting point from where more sophisticated generalizing models such as mixed logit may be applied.

A DCE model specifies the probability that an individual will choose a specific smoking cessation app. This probability is expressed as a function of measured attributes specific to the alternative. The (simplified) underlying utility function for alternative *j* is shown in equation 1:

utility of option *j* = β1(alternative1) + β2(opt-out) + β3(developer_not_shown) + β4(developer_NHS) + β5(ratings4.8_stars) + β6(ratings4_stars) + β7(ratings3.2_stars) + β8(descriptionsShort) + β9(descriptionsLong) + β10(imagesScreenshots)+ β11(imagesBoth) + β12(costs_in_£) + ε **(1)**

In equation 1, *U* represents the overall utility gained from choosing alternative *j*, β is the mean coefficient attached to the corresponding attribute levels estimated by the mixed logit model and represents the part-worth utility attached to each attribute level, and ε is the random error of the model. β1 and β2 represent alternative specific constants.

Our DCE included three attributes (A) with three levels (L) and two attributes with four levels, which, following the formula L^A^ would have led to 432 possible choice alternatives in a full factorial design [28]. To limit participant burden, we used a fractional factorial design. We generated 48 choice tasks applying Bayesian D-efficient design principles using Ngene software (ChoiceMetrics) [29] and blocked them into four survey versions each containing 12 choice tasks (Multimedia Appendix 2). The choice tasks in each block and the order of the attributes were fixed. Each participant was randomized to complete one survey. An additional repeat choice task was added to test choice consistency which was excluded from the primary data analysis. This means one task was repeated which allowed us to examine the percentage of people who consistently chose the same alternative This design aimed to estimate the main effects and assumes no interaction between attributes in the design.

The initial version of the DCE was piloted web-based with 49 participants. During the pilot phase, the understanding of the tasks was explored, and feedback on the wording of the attributes was sought. Based on the feedback received, the wording of the survey and the order in which attributes were listed in the table were revised. Coefficients from the pilot phase were used as priors to estimate a Bayesian D-efficient design. Data from the pilot phase were not included in the final analysis.

**Figure 1:**
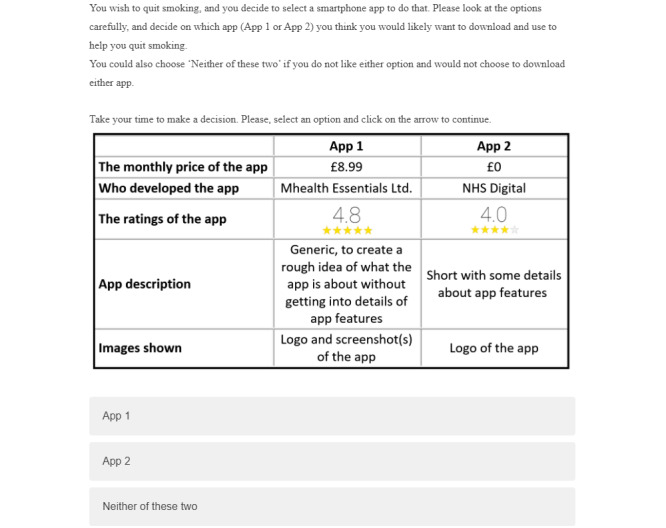
Example of a choice task with an opt-out option in the discrete choice experiment to explore the uptake of a smoking cessation app among adult smokers in the United Kingdom between December 2020 and February 2021.

To imitate real-world decisions regarding app uptake, an opt-out option was included (“Neither of these two”; Figure 1). Participants who chose the opt-out option were prompted to repeat the decision and make a forced choice between the two alternatives. As the rate of the opt-out was low, the complete dataset was used for the analysis of choice data, including the opt-out option.

### Data Collection

#### Participants and Recruitment

This study was conducted digitally. Eligible participants were adults (1) aged 18 years and older, (2) residents in the United Kingdom, (3) able to give consent, (4) owned or had primary use of a smartphone, (5) smoked cigarettes, and (6) interested in quitting smoking using a smartphone app. Recruitment took place between December 2020 and February 2021, through social media, the “Call for Participants” platform, and the Prolific platform. Recruitment posts were shared on Facebook, Twitter, and LinkedIn to reach potential participants. This study was advertised on the “Call for Participants” platform as well. Participants recruited through these channels accessed a link to the survey in Qualtrics (Qualtrics International Inc) that included a screening questionnaire to determine eligibility. Those who met the criteria were able to complete the survey.

Prolific is a crowdsourcing platform that connects researchers with individuals willing to participate in studies. Prolific enabled efficient and targeted recruitment without the need for advertising. Since Prolific screened participants based on specified criteria, eligible individuals were redirected to a second version of the Qualtrics survey that contained only the survey questions, without the additional screening.

#### Sample Size

The sample size calculation was based on a rule-of-thumb formula (equation 2) [30].

N>500c/(*t*×*a*) **(2)**

In equation 2, “N” represents the sample size, *t* the number of tasks per participant (=12), “*a*” the number of alternatives in each choice task (=2), “*c*” the number of analysis cells (=4, as this is the largest number of levels for any of the attributes). Equation 2 suggests a minimum sample size of 83. With four versions of the survey, we targeted a sample size of at least 332 (4×83) participants.

### Procedure

The survey was administered using Qualtrics survey software. Once consent was obtained participants were explained the DCE and were randomly assigned to one of the four DCE versions containing 13 choice tasks (12 from the design plus one repeat choice task) and were asked to complete further measures (see *measures*).

### Measures

#### Attributes That Are Likely to Influence Smoking Cessation App Preferences

The primary outcomes are the marginal effects estimated for each attribute level in the choice model, represented by the β coefficients in equation 1.

#### Factors Perceived to Influence Uptake and Engagement With the Smoking Cessation App

We used the TDF to identify 13 potential facilitators and barriers to uptake and engagement with health apps based on factors identified as important in our previous work [7,10]. These were included in the survey as a set of statements with the level of agreement with the statements measured using a 5-point Likert-type scale. For analysis, responses to these statements were dichotomized into agree (strongly agree and agree) versus not (neither agree nor disagree, disagree, and strongly disagree; Multimedia Appendix 2).

#### Other Variables

The survey included questions about the previous use of smoking cessation apps and other health apps, user type (power user or minimal user) based on preferences exploring app features (ie, whether individuals use basic features and spend the minimum amount of time navigating the app [minimal users] versus those who would enjoy spending time navigating through features and engaging with the app regularly [power users]). Smoking behavior measures were heaviness of smoking index [31], frequency of smoking, attempts to stop smoking, strategies used in an attempt to quit smoking, intention to stop smoking, defined as whether the participant is planning to quit in the next 6 months, determination to stop smoking, and the main reason for stopping smoking. Sociodemographic characteristics were also measured (age, gender, level of education, household income, ethnicity, sexuality, and disability). See Multimedia Appendix 2 for the complete questionnaire.

### Statistical Analysis

The pilot data were analyzed using the Apollo package in R (R Core Team; programming language developed by the R Foundation for Statistical Computing) [32], and the final data using Stata (version 16.1; StataCorp LLC). Participants’ characteristics and the TDF factors perceived to influence the engagement with smoking cessation apps were summarized using descriptive statistics. Associations between attributes and uptake responses were estimated using a mixed logit model (MIXL). This approach accommodated the existence of preference heterogeneity within the sample by allowing one or more model parameters to be specified as having a random distribution [33]. In the model, all attributes were dummy-coded as categorical variables, except for cost, which was treated as continuous after verifying a linear relationship with utility, and all were treated as normally distributed random parameters. The model allowed for correlation between parameters. We analyzed data of participants who completed the full survey. The model was fit using 2000 Halton draws. We investigated the uptake of the most preferred and least preferred apps by calculating their utility values and the probabilities for selecting these hypothetical apps, using the approach described by Jonker et al [34]. To facilitate willingness to pay (WTP) estimation, the same model was used but specified cost as a fixed parameter. We analyzed the choice data of participants who were consistent with the repeat choice task and compared it to the results of the choice data including all participants. As part of a post hoc analysis poolability of the two different samples (incentivized Prolific sample; nonincentivized “other” sample), was assessed following the Swait and Louviere [35] procedure. The TDF factors perceived to influence the engagement with smoking cessation apps were described using proportions and 95% CIs.

### Ethical Considerations

Ethical approval for this study was obtained from the University of East Anglia Faculty of Health Ethics Committee (reference: 2020/21-017). This approval covered all aspects of participant recruitment, data collection, and analysis. The study protocol was preregistered on the Open Science Framework [36]. Participants recruited on Prolific were paid US $1.90 (UK £1.50) for participation and those recruited on other platforms were invited to participate in a prize draw to win one of ten US $25.36 (UK £20) shopping vouchers. Participants provided informed consent before beginning the survey. Consent was obtained through Qualtrics survey software from participants who met the eligibility criteria, where participants were presented with an information sheet and consent form prior to accessing the DCE and additional questions. Participant data were anonymized to protect privacy. No identifiable information was collected, and all data were stored securely in accordance with institutional data protection policies. Responses were deidentified before analysis to ensure confidentiality.

## Results

A total of 499 eligible participants were recruited, 469 participants consented, and 337 participants completed the experiment and measures. Out of these 337 participants, 196 participants were recruited through the Prolific website, and the rest through social media and through the Call for Participants website. Data from 337 participants yielded 4029 observations (15 choices were omitted by participants). Participants were aged between 19 and 65 years, with a mean age of 35 (SD 11) years, 168 (49.8%) were female participants, 176 (52.2%) participants showed low dependency on the heaviness of smoking index, and 107 (31.8%) participants had used smoking cessation apps before. Participants’ characteristics are reported in Table 2.

**Table 2:** Characteristics of study participants in DCE^a^ conducted between December 2020 and February 2021 to explore preferences for smoking cessation apps among adult smokers in the United Kingdom.

Sociodemographic data		Value (N=337)		
**Age (years)**				
	Range		19-65	
	Mean (SD)		35 (11)	
**Sex,** **n** **(%)**				
	Female		168 (49.8)	
	Male		163 (48.4)	
	Nonbinary or genderfluid		4 (1.2)	
	Prefer not to disclose		2 (0.6)	
**Ethnicity,** **n** **(%)**				
	Arab		10 (3)	
	Asian		6 (1.8)	
	Black or African American		11 (3.2)	
	Mixed or multiple ethnic groups		8 (2.4)	
	White		300 (89)	
	Other		2 (0.6)	
**Education,** **n** **(%)**				
	Postgraduate or equivalent		31 (9.2)	
	Degree or equivalent		127 (37.7)	
	A-levels or equivalent		113 (33.5)	
	GSCE^b^ or equivalent		63 (18.7)	
	Other		3 (0.9)	
**Monthly net household income,** **n** **(%)**				
	US $0-$1267 (UK £0-£999)		39 (11.6)	
	US $1268-$2535 (UK £1000-£1999)		112 (23.2)	
	US $2536-$3803 (UK £2000-£2999)		68 (20.2)	
	US $3804-$5071 (UK £3000-£3999)		48 (14.2)	
	US $5072-$6339 (UK £4000-£4999)		23 (6.9)	
	Over US $6340 (UK £5000)		15 (4.4)	
	Prefer not to disclose		32 (9.5)	
**Sexual orientation,** **n** **(%)**				
	Heterosexual		268 (79.5)	
	LGBTQ+^c^		64 (19)	
	Prefer not to say		5 (1.5)	
**Disability,** **n** **(%)**				
	Living with disability		88 (26.1)	
	No disability		232 (68.8)	
	Prefer not to disclose		17 (5.1)	
**Type of smartphone,** **n** **(%)**				
	Android		163 (48.4)	
	Apple		164 (48.6)	
	Android and Apple		8 (2.4)	
	Windows		2 (0.6)	
**Prior use of health app,** **n** **(%)**				
	Prior use of health app		226 (67.1)	
	No prior use of health app		111 (32.9)	
	Prior use of smoking cessation app		107 (31.8)	
	No prior use of smoking cessation app		230 (68.2)	
**Health app uptake source^d^, n (%)**				
	Google search		62 (25.7)	
	Commercial app stores		158 (65.6)	
	Health-related website		51 (21.2)	
	Recommendations (friends, family)		58 (24.1)	
	Recommendations (health practitioners)		21 (8.7)	
	Other		6 (2.5)	
**User type^d^, n (%)**				
	Power user		113 (46.9)	
	Minimal user		120 (49.8)	
	Unsure		8 (3.3)	
**Heaviness of smoking^e^, n (%)**				
	Low dependence		176 (52.2)	
	Moderate dependence		139 (41.3)	
	High dependence		22 (6.5)	
**Last quit attempt,** **n** **(%)**				
	In the last month		44 (13.1)	
	In the last 12 months		136 (40.3)	
	Longer than 12 months		113 (33.5)	
	None		44 (13.1)	
**Previous experience with smoking cessation strategies,** **n** **(%)**				
	Nicotine replacement products		148 (43.9)	
	Zyban (buprorion)		9 (2.7)	
	Champix (varenicline)		26 (7.7)	
	E-cigarette or vaping device		195 (57.9)	
	Stop smoking group		28 (8.3)	
	Stop Smoking one-to-one counseling or support services		35 (10.4)	
	Smoking helpline		16 (4.8)	
	A book about quitting smoking		51 (15.1)	
	Smoking cessation website		54 (16)	
	Smoking cessation app		59 (17.5)	
	Other: hypnotherapy		2 (0.6)	
	None		66 (19.6)	
**Intention to quit in the next 6 months,** **n** **(%)**				
	Likely		240 (71.3)	
	Unlikely		23 (6.8)	
	Unsure		74 (21.9)	
**Determination to quit,** **n** **(%)**				
	High determination		216 (64.1)	
	Moderately or slightly determined		113 (33.5)	
	Low determination		8 (2.4)	
**Main reason to quit,** **n** **(%)**				
	Health concerns		125 (37.1)	
	Health concerns related to COVID-19		28 (8.3)	
	To save money		112 (33.2)	
	To regain control		42 (12.5)	
	Pressure or encouragement from others		27 (8)	
	Other		3 (0.9)	

^a^DCE: discrete choice experiment.

^b^GSCE: General Certificate of Secondary Education.

^c^LGBTQ+: lesbian, gay, bisexual, transgender, and queer or questioning.

^d^Questions answered by those who have used smoking cessation or health apps before.

^e^Computed from number of cigarettes smoked a day and the time the first cigarette is smoked in the morning [31].

On 89.9% of the choices, participants selected one of the two smoking cessation apps over “neither.” There was no participant who opted out of all choices. Most of the attributes influenced participants’ preferences, except for the description of the app (Table 3). Relative to other attributes and given the levels included in the DCE, social proof (the star rating of the app) was the most important attribute. Relative to the referent app (developed by Mhealth Essentials, star rating not shown, generic app description, with a logo shown only), having a 4.8-star rating (mean preference parameter 2.27, SD 1.55; 95% CI 1.95-2.59) was around twice as important as the 4-star rating (mean preference parameter 1.06, SD 1.32; 95% CI 0.78-1.34), and twice as important as having a credible source (it being developed by the NHS Digital, mean preference parameter 0.93, SD 1.23; 95% CI 0.72-1.15). Participants marginally preferred an app that showed screenshots (mean preference parameter 0.35, SD 0.50; 95% CI 0.19-0.52) or both screenshot and logo (mean preference parameter 0.39, SD 0.96; 95% CI 0.19-0.59) over logo only. An app with a low monthly price was also preferred (mean preference parameter –0.38, SD 0.33; 95% CI –0.44 to –0.32). However, the wide SDs, relative to their coefficients for many attributes indicate a broad variation in attribute importance among participants. There was significant preference heterogeneity across all attribute levels.

The characteristics of the most preferred app were having a monthly cost of US $0 (UK £0), a rating of 4.8 stars, developed by NHS Digital, having a generic description, and presenting both types of images (app logo and screenshots). The least preferred app has a monthly price of US $11.40 (UK £8.99), the developer is not shown, ratings of 3.2 stars, a long description, and shows the app logo only. The uptake level of the best app was estimated at 95%, and for the worst was estimated at 10%.

Table 4 reports marginal WTP estimates for improvement in the attributes of the app, relative to the reference category. Participants were willing to pay an additional US $6.96 (UK £5.49; 95% CI $6.15 [£4.85]-$7.75 [£6.11]) and US $3.58 (UK £2.82; 95% CI $2.89 [£2.28]-$4.26 [£3.36]) for app with 4.8- and 4-star ratings, respectively. Participants were willing to pay US $2.61 (UK £2.06; 95% CI $2.03 [£1.60]-$3.20 [£2.52]) for development by a trusted organization (NHS Digital) compared to Mhealth Essentials Ltd.

A total of 71 (21%) individuals were inconsistent with their choices. The demographics of this group were similar to those who were consistent with their choices. The results of the MIXL model with and without the individual’s response who gave an inconsistent response to the repeat choice task returned comparable results (data not presented, available from author). However, in a post hoc analysis to investigate whether the preference parameters of the two sample groups were equal, strong evidence was found to reject this hypothesis (*P*<.001), indicating different choice preferences.

Participants indicated that the strongest facilitators that might promote their engagement with a smoking cessation app were user guidance of how to use the app (72.4% agreement, 95% CI 67.37%-76.93%), additional health information (75% agreement, 95% CI 70.16%-79.42%), and rewards (75.4% agreement, 95% CI 70.47%-79.69%; Table 5). Key barriers were concerns around data protection (66.8% agreement, 95% CI 61.54%-71.61%) cognitive load (47.5% agreement, 95% CI 42.16%-52.87%), reminders as triggers for cravings (40.7% agreement, 95% CI 35.51%-46%), and peer support (46.9% agreement, 95% CI 41.59%-52.25%).

**Table 3:** Mixed logit estimation results^a^ from the DCE^b^ exploring preferences for smoking cessation apps among adult smokers in the United Kingdom (December 2020-February 2021).

Attributes				Mean preference parameter (SE)		95% CI
**Alternative specific constants**						
	**Alternative 1**					
		Mean	0.15^c^ (0.06)		0.02 to 0.27	
		SD^d^	0.14 (0.09)		–0.03 to 0.31	
	**Alternative 2**					
		Mean	Reference		N/A^e^	
		SD	Reference		N/A	
	**Opt out option**					
		Mean	–2.08^f^ (0.22)		–2.50 to –1.65	
		SD	1.77^f^ (0.23)		1.31 to 2.23	
**Developer**						
	**Does not say**					
		Mean	–0.48^f^ (0.10)		–0.68 to –0.27	
		SD	0.84^f^ (0.13)		0.58 to 1.11	
	**Mhealth Essentials**					
		Mean	Reference		N/A	
		SD	Reference		N/A	
	**NHS Digital**					
		Mean	0.93^f^ (0.11)		0.72 to 1.15	
		SD	1.23^f^ (0.14)		0.96 to 1.50	
**Rating of the app**						
	**Does not show**					
		Mean	Reference		N/A	
		SD	Reference		N/A	
	**4.8 stars**					
		Mean	2.27^f^ (0.16)		1.95 to 2.59	
		SD	1.55^f^ (0.17)		1.21 to 1.89	
	**4 stars**					
		Mean	1.06^f^ (0.14)		0.78 to 1.34	
		SD	1.32^f^ (0.18)		0.96 to 1.68	
	**3.2 stars**					
		Mean	0.33^c^ (0.14)		0.04 to 0.61	
		SD	1.65^f^ (0.18)		1.30 to 1.99	
**App description**						
	**Generic**					
		Mean	Reference		N/A	
		SD	Reference		N/A	
	**Short**					
		Mean	–0.02 (0.08)		–0.19 to 0.15	
		SD	0.50^f^ (0.13)		0.25 to 0.75	
	**Long**					
		Mean	–0.19 (0.11)		–0.41 to 0.03	
		SD	1.17^f^ (0.14)		0.90 to 1.44	
**Images**						
	**Logo**					
		Mean	Reference		N/A	
		SD	Reference		N/A	
	**Screenshot**					
		Mean	0.35^f^ (0.08)		0.19 to 0.52	
		SD	0.50^f^ (0.12)		0.26 to 0.75	
	**Both**					
		Mean	0.39^f^ (0.10)		0.19 to 0.59	
		SD	0.96^f^ (0.13)		0.73 to 1.26	
**Monthly price of the app^g^**						
	Mean			–0.38^f^ (0.03)		–0.44 to –0.32
	SD			0.33^f^ (0.03)		0.27 to 0.41

^a^Akaike information criterion=6294.02; Bayesian Information criterion=6960.01; Log-likelihood=–3057.01.

^b^DCE: discrete choice experiment.

^c^*P* value of <.05.

^d^SD of the distribution around the mean preference estimate and is a measure of heterogeneity.

^e^Not applicable.

^f^*P* value of <.001.

^g^The monthly price of the app was coded as a continuous variable presented at four levels: US $0 (UK £0), US $3.79 (UK £2.99), US $7.60 (UK £5.99), and US $11.40 (UK £8.99).

**Table 4:** Willingness to pay results from the DCE^a^ exploring preferences for smoking cessation apps among adult smokers in the United Kingdom (December 2020-February 2021).

Attribute	Acceptable reduction in price, WTP^b^ (SE)	95% CI
App with a rating of 4.8 stars	US $6.69 (US $0.41); £5.49 (£0.32)	US $6.15 to US $7.75 (£4.85 to £6.11)
App with a rating of 4 stars	US $3.58 (US $0.34); £2.82 (£0.27)	US $2.89 to US $4.26 (£2.28 to £3.36)
App with a rating of 3.2 stars	US $0.41(US $0.38); £0.32 (£0.30)	US $–0.36 to US $1.15 (£–0.28 to £0.91)
App developed by the NHS^c^	US $2.61(US $0.29); £2.06 (£0.23)	US $2.03 to US $3.20 (£1.60 to £2.52)
App developer not shown	US $–1.56 (US $0.28); £–1.23 (£0.22)	US $–2.09 to US $–1.01 (£–1.65 to £–0.80)
App showing logo and screenshots	US $0.96 (US $0.27); £0.76 (£0.21)	US $0.43 to US $1.50 (£0.34 to £1.18)
App showing screenshots only	US $0.75 (US $0.23); £0.59 (£0.18)	US $0.30 to US $1.19 (£0.24 to £0.94)
App presented with a short description	US $–0.24 (US $0.23); £–0.19 (£0.18)	US $–0.70 to US $0.20 (£–0.55 to £0.16)
App presented with a long description	US $–0.06 (US $0.29); £–0.05 (£0.23)	US $-0.63 to US $0.51 (£–0.50 to £0.40)

^a^DCE: discrete choice experiment.

^b^WTP: willingness to pay.

^c^NHS: National Health Service.

**Table 5:** Percentage of potential factors influencing smokers’ uptake and engagement with smoking cessation apps, as identified in a survey conducted among adult smokers in the United Kingdom^a^.

				Percentage, %		95% CI
**TDF^b^ construct: Skills**						
	**App literacy (facilitator): In general, I can easily use a newly installed app on my phone**					
		Agree	92.6		89.24-94.95	
**TDF construct: Knowledge**						
	**App awareness (barrier): I was aware of the existence of smoking cessation apps prior to taking part in this study**					
		Agree	55.5		50.12-60.73	
	**User guidance (facilitator): A guide of how to use features would help me use the app more often**					
		Agree	72.4		67.37-76.93	
	**Health information (facilitator): Information in the app about how quitting smoking improves my health would make me use the app more often**					
		Agree	75		70.16-79.42	
**TDF construct: Memory, attention, decision processes**						
	**Cognitive load** **(barrier)** ***:*** **In general, I don’t want to use an app with features that would take some time to learn**					
		Agree	47.5		42.16-52.87	
	**Reminders (facilitator): It would be important that an app to help me quit smoking sends personalised reminders to me**					
		Agree	68.3		63.07-73.02	
	**Reminders (barrier): I wouldn’t want to use an app that sent me reminders about quitting smoking in case it would trigger my cravings to smoke**					
		Agree	40.7		35.51-46	
**TDF construct: Social influence**						
	**Peer-support (facilitator): Being connected with other app users would motivate me to stay on track with my intention to stop smoking**					
		Agree	65.6		60.32-70.48	
	**Peer-support (barrier): Being connected with other app users would make me feel ashamed or disappointed if I started smoking again after quitting**					
		Agree	46.9		41.59-52.25	
	**Professional support (facilitator): Being connected with online helpers (quit smoking advisors) within the app would make want to use the app more**					
		Agree	69.5		64.29-74.14	
**TDF construct: Beliefs about capabilities**						
	**Self-confidence (facilitator): I am confident I could quit smoking by using an app**					
		Agree	50.7		45.4-56.07	
**TDF construct: Beliefs about consequences**						
	**Data protection (barrier): I am concerned how my personal data is handled in apps**					
		Agree	66.8		61.54-71.61	
**TDF construct: Goals**						
	**Goal setting and action planning: Receiving guidance of how to achieve goals is more important for me than just simply setting goals**					
		Agree	84.3		79.97-87.79	
**TDF construct: Social identity**						
	**Social identity (barrier): When using a smoking cessation app, I don’t want to feel that I am being treated like a patient**					
		Agree	61.4		56.1-66.49	
**TDF construct: Reinforcement**						
	**Rewards (facilitator): Receiving badges or awards for achieving a set goal, would make me use the app more often**					
		Agree	75.4		70.47-79.69	

^a^Factors were derived using the theoretical domains framework and measured through participant responses collected between December 2020 and February 2021.

^b^TDF: theoretical domains framework.

## Discussion

### Main Findings

This study investigated five potential attributes each mapped to domains from the TDF, potentially relevant to the uptake of smoking cessation apps. Participants made choices between hypothetical app alternatives with predefined attributes: (1) monthly price (environmental resources), (2) developer credibility (social influence, as a credible source), (3) app rating (social influence, as social proof), (4) app description (beliefs about consequences), and (5) images (beliefs about consequences).

Participants preferred apps demonstrating strong social proof (high star ratings) and a credible source (trusted organizations as developers), along with a lower monthly cost. The description of the app shown to participants did not influence preferences.

Relative to other attributes, social proof (a high star rating) emerged as a particularly influential factor, consistent with findings from previous studies [7,17]. Familiarity with highly rated apps may contribute to this preference, as these apps are likely to appear at the top of search lists. However, not all high-quality evidence-based smoking cessation apps have high star ratings [16,18], which suggests that social proof may influence uptake more than evidence-based content.

The preference for credible sources, in this case, apps from trusted organizations, such as NHS Digital, aligns with existing evidence. Our findings are similar to a DCE that investigated the uptake of a COVID-19 tracing app in the United Kingdom where participants were more likely to adopt an NHS contact tracing app [37]. This reflects broader user concerns about reputable sources [38] and a preference for apps developed by experts over those from unknown or less reputable sources [39].

Interestingly, descriptions, however, did not seem to influence the uptake of a smoking cessation app. A plausible explanation is that this attribute may not have been described or presented in a way to capture the participants’ attention. To avoid cognitive load, we provided brief, verbal definitions for generic, short, or long descriptions rather than examples. This presentation choice may have limited the salience of the app description attribute in this study, potentially impacting its relevance to participants.

Consistent with similar studies, our findings suggest that participants most preferred apps with no cost [10,40]. However, some individuals indicated a WTP for an app if it offers features aligned with their preferences, such as being developed by a credible source [7]. Preliminary estimates of marginal WTP suggest that users may accept a small fee if the app demonstrates strong social proof or is from a trusted organization, though these estimates should be interpreted with caution.

Only around half of our participants were aware of smoking cessation apps, indicating that further awareness efforts could help make these tools more accessible. In line with previous findings, access to health information and a user guide to using the app were viewed as factors that could improve engagement [7,10], particularly among participants with limited app literacy. Less than half of the participants reported they would not want to use an app with complex features. We previously found mixed views on reminders, with some believing they may negatively influence behavior change by triggering cravings [7,10]. In this study, we found that less than half of the participants reported reminders as barriers. Consistent with previous findings [7,10], potential users believed that peer and professional support could increase engagement [7,10], and fewer than half indicated that failure to quit would lead to disappointment. This shows the difficulty app developers may face when developing an app to suit most individuals’ needs and the potential importance of guidance from organizations such as the UK National Institute of Health and Care on developing digital behavior change tools.

Overall, our findings suggest that social proof (star ratings) and credible source (apps developed by trusted organizations) may be more important for individuals than the price of the app. Additionally, we investigated additional factors related to engagement, mapped under the TDF. Together, these findings offer preliminary insights into user preferences that may be considered in app presentations on curated health app portals.

### Implications

The findings of this study offer preliminary insights that may assist public health organizations in increasing the uptake of evidence or theory-informed smoking apps. They could also guide health app providers and curators of health app portals, such as the NHS Apps Library, in optimizing app presentation to better align with user preferences, potentially enhancing app uptake within curated health platforms.

App uptake was found to be driven primarily by price and social influence (app ratings), while presentation factors, such as the app’s description and visual elements, were found to have a limited influence on choice, with a combined relative importance of under 8%. This relatively low level of importance ascribed to presentation may be partly due to the generic descriptions of these attributes used in the experiment, which may not have captured participants’ attention or preferences fully.

While the information presented may help app curators prioritize specific information, such as cost and user ratings, when presenting apps, the scope of this study is limited to uptake drivers and does not extend to factors influencing ongoing user satisfaction or engagement.

The WTP estimates quantify the relative preference of smokers for the attributes in an accessible format. However, these values should be interpreted with caution, as they represent initial estimates that require further validation before being applied more widely.

The integration of the TDF in this study aimed to provide a structured overview of attributes influencing app choice, building on previous research where the TDF was similarly applied. While these findings complement prior qualitative data [7,41] and systematic review [10], further research is warranted to establish a more comprehensive understanding of how individual app features impact social proof (user ratings), quality perceptions, and long-term engagement.

### Limitations

Although the recruitment was designed to include a wide range of participants, the sampling strategy used is unlikely to have generated a representative sample of smokers. Some views may have been missed by recruiting exclusively digitally, including views of individuals experiencing homelessness, those living in deprived areas, and those living in areas without suitable internet coverage.

The design of this study investigated main effects only, therefore, possible interactions between attributes were not assessed. The sample size was inadequate to enable investigating stratifications of certain demographics. The DCE was piloted and refined based on feedback but the clarity and usability, ease of completion, or understanding of the DCE were not further assessed in the main study, for example, no think-aloud cognitive pilot interviews were conducted. While respondents were screened based on willingness to use an app to aid quitting, prompting participants to make a forced choice when they chose the opt-out option might have influenced their choice behavior. An example of this might be the avoidance of selecting the opt-out, in anticipation of the forced choice question, however, opt-out choices were evenly distributed across the 12 choices, indicating that this is not the case.

Prompting participants to make a forced choice when they chose the opt-out option might have influenced their choice behavior and in anticipation of the forced choice question, they may have chosen an alternative throughout the survey, which could be a reason for the small number of cases where the opt-out option was chosen Additionally, using a hypothetical company may have influenced smokers’ preferences (in either positive or negative direction), compared to if a real company was used.

This study investigated the uptake of a smoking cessation app based on stated preferences, which may be different from the uptake of a smoking cessation app in real life. This study focused primarily on the uptake of smoking cessation apps and did not consider all previously identified uptake and engagement factors that may shape choice behavior (eg, the aesthetics of the app, reminders, social networking, and embedded health professional support [7,10]), which limits our conclusions about the relative importance of the factors studied. Additionally, this study included one attribute—user ratings—that indirectly reflected app quality and functionality. Future studies may benefit from including further attributes that indicate other quality parameters.

The specification of our model also introduces limitations. By specifying a normal distribution for cost in the main model, 12.5% of respondents are predicted to have positive price parameter. Behaviorally, this seems counterintuitive, however, some people may prefer a paid app over a free app (eg, because they associate cost with quality). Second, in order to facilitate a straightforward estimation of WTP, we specify cost as a fixed parameter. This is of course inconsistent with the results from the main model, which indicate significant heterogeneity with regards to preference for the cost of the app. This approach has the potential to severely bias WTP estimates, which should consequently be interpreted with caution.

The results on the poolability of the sample indicate that pooling of the 2 samples (those recruited from the incentivized Prolific site, and those recruited from elsewhere) was not appropriate, as there were substantial differences between how the independent variables influenced choice in each group. Despite highly similar demographic characteristics between the 2 groups, choice behavior may have been influenced by incentivization or other, unobserved differences between the 2 groups, biasing our results.

The relative importance of the attributes may vary between genders and age groups. Future DCEs may want to consider recruiting a larger sample size to investigate the relative importance of the attributes stratified based on sociodemographical factors. Knowing the preferences of certain groups with specific demographics may help to target the presentation of apps to increase uptake. To build on our limited conceptualization of the perceived utility of the app, future DCEs could borrow ideas from interaction design and user research studies and apply a visual representation of apps, instead of textual description. In this case, participants are shown images of apps as opposed to a table where logos are visually represented as opposed to described. Similarly, “app descriptions” could include typical feature content presented in different ways rather than abstractly reflecting different types of description (generic, short, or long). Finally, the impact of health apps is a combination of uptake and effectiveness. The measured factors influencing the uptake and engagement with smoking cessation apps suggest that more empirical studies, including testing in real-world situations, are needed to fully understand the extent of facilitators and barriers. There is very limited opportunity to investigate the external validity of this study, but it is hoped that as technology advances, opportunities will arise and test this research in real-life settings.

### Conclusions

This study found that uptake is more likely if smoking cessation apps demonstrate strong social proof (high star ratings), are developed by a credible source (trusted organization), include screenshots, and are low cost. However, star ratings showed a relatively stronger influence on app selection compared to other attributes within the range of rating scores investigated.

## Appendix

Multimedia Appendix 1 Checklist for reporting discrete choice experiments in health: The DIRECT Checklist.

Multimedia Appendix 2 The choice sets and full survey questionnaire.

## Abbreviations

DCE: discrete choice experiment
DiGA: Digital Health Applications
DIRECT: Discrete Choice Experiment Reporting Checklist
NHS: National Health Service
TDF: theoretical domains framework
WTP: willingness to pay

## Data Availability

The dataset generated during this study is available on Open Science Framework [36].
