# Supplementary material for: Eliciting Preferences for the Uptake of Smoking Cessation Apps: Discrete Choice Experiment
Source: J Med Internet Res. 2025 Jan 14;27:e37083. doi: 10.2196/37083 (PMC11775483; doi:10.2196/37083)
Supplement: Multimedia Appendix 1 [file jmir_v27i1e37083_app1.docx]

# Checklist for reporting discrete choice experiments in health: The DIRECT Checklist*

| **Section Item** | | **Page** |
| --- | --- | --- |
| Purpose and rationale | |  |
| 1 | Describe the real-world context and decision-maker that the hypothetical choice context seeks to replicate or inform | Page 2; |
| 2 | Provide a rationale for using a DCE to answer the research question | Page 2 |
| Attributes and levels^a^ | |  |
| 3 | Describe how attributes and levels were derived (e.g. literature review, interviews, focus groups, expert input) | Page 2 |
| 4 | Provide the final list of attributes and levels | Page 3 |
| Experimental design | |  |
| 5 | Report the number of alternatives per choice set and whether they were labelled or unlabelled | Page 3 |
| 6 | Describe response options (e.g. forced choice, opt-out, status quo) | Page 3 |
| 7 | Describe the type of experimental design (e.g. orthogonal, D-efficient, Bayesian efficient, partial profile) | Page 3 |
| 8 | Describe which effects are identified in the design (e.g. main effects, higher order interactions, functional form) | Page 3 |
| 9 | Describe the number of choice sets, blocks and choice sets per block | Page 3 |
| 10 | Indicate how the experimental design was obtained (software, catalogue, other) | Page 3 |
| Survey design | |  |
| 11 | Provide a sample choice set and the instructions and background information given to respondents (e.g. providing the survey as an appendix) | Page 4, Figure 1, Appendix 2 |
| 12 | Report any randomisation (e.g. choice set order, attribute order, alternative order, framing effects) | Page 3 |
| 13 | Describe what was checked in piloting (e.g. understanding, respondent burden, timing, wording) | Page 3 |
| 14 | Report whether information from the pilot was used to update the experimental design (e.g. priors, functional form of attributes) or survey design | Page 3 |
| Sample and data collection | |  |
| 15 | Report respondent inclusion/exclusion criteria | Page 4 |
| 16 | Describe how data were collected (e.g. mail, personal interview, web survey) | Page 4 |
| 17 | Report the response rate or cooperation rate, if possible | Page 5 |
| 18 | Report the final sample size and how the sample size was determined | Page 4 |
| 19 | Describe respondent characteristics and representativeness of target population, if known | Page 6, Table 2 |
| Econometric analysis | |  |
| 20 | Indicate coding of data (e.g. effects, dummy, continuous) including definitions | Page 3 |
| 21 | Report whether any respondents were removed and why (e.g. suspected fraudulent responses, rationality tests) | n/a |
| 22 | Provide the rationale for model choice (e.g. conditional logit, mixed logit, latent class) and assumptions (e.g. error variance) | Page 3 |
| 23 | Report model specification | Page 3 |
| Reporting of results | |  |
| 24 | Report the model performance, goodness of fit (if comparing models) | Page 10, footnote in Table 3 |
| 25 | Describe methods used for analysis of model results (e.g. calculation of marginal rate of substitution, attribute relative importance, welfare gain) | Page 5 |
| 26 | Report measures of precision for the output(s) of interest (e.g. confidence intervals) and how these were derived | Page 10 Table 3, Page 11 Table 4 |

*Ride J, Goranitis I, Meng Y, LaBond C, Lancsar E. A Reporting Checklist for Discrete Choice Experiments in Health: The DIRECT Checklist. Pharmacoeconomics. 2024 Oct;42(10):1161-1175. doi: 10.1007/s40273-024-01431-6. Epub 2024 Sep 3. PMID: 39227559; PMCID: PMC11405421.
