# Supplementary material for: Eliciting Preferences for the Uptake of Smoking Cessation Apps: Discrete Choice Experiment
Source: J Med Internet Res. 2025 Jan 14;27:e37083. doi: 10.2196/37083 (PMC11775483; doi:10.2196/37083)
Supplement: Multimedia Appendix 2 [file jmir_v27i1e37083_app2.docx]

1. **The 48 choice sets generated in 4 blocks**

**Block 1**

**Choice situations: 4, 9, 10, 15, 18, 22, 29, 32, 33, 34, 38, 47**

**Scenario 4 Block 1**

|  | **App 1** | **App 2** |
| --- | --- | --- |
| App description | Long and detailed description of the app and its features | Short with some details about app features |
| The ratings of the app | 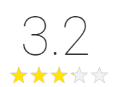 | Does not show |
| Images shown | Screenshot(s) of the app | Logo and screenshot(s) of the app |
| Who developed the app | Does not show | Mhealth Essentials Ltd. |
| The monthly price of the app | £0 | £0 |

**Scenario 9 Block 1**

|  | **App 1** | **App 2** |
| --- | --- | --- |
| App description | Long and detailed description of the app and its features | Short with some details about app features |
| The ratings of the app | 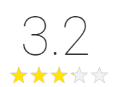 | 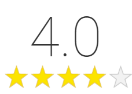 |
| Images shown | Logo of the app | Screenshot(s) of the app |
| Who developed the app | Does not show | NHS Digital |
| The monthly price of the app | £2.99 | £5.99 |

**Scenario 10 Block 1**

|  | **App 1** | **App 2** |
| --- | --- | --- |
| App description | Generic, to create a rough idea of what the app is about without getting into details of app features | Long and detailed description of the app and its features |
| The ratings of the app | 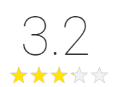 | Does not show |
| Images shown | Logo and screenshot(s) of the app | Screenshot(s) of the app |
| Who developed the app | Does not show | Mhealth Essentials Ltd. |
| The monthly price of the app | £5.99 | £8.99 |

**Scenario 15 Block 1**

|  | **App 1** | **App 2** |
| --- | --- | --- |
| App description | Long and detailed description of the app and its features | Generic, to create a rough idea of what the app is about without getting into details of app features |
| The ratings of the app | Does not show | 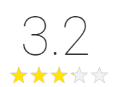 |
| Images shown | Logo of the app | Logo and screenshot(s) of the app |
| Who developed the app | Does not show | NHS Digital |
| The monthly price of the app | £8.99 | £8.99 |

**Scenario 18 Block 1**

|  | **App 1** | **App 2** |
| --- | --- | --- |
| App description | Short with some details about app features | Generic, to create a rough idea of what the app is about without getting into details of app features |
| The ratings of the app | 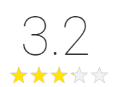 | 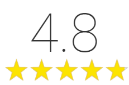 |
| Images shown | Logo of the app | Screenshot(s) of the app |
| Who developed the app | NHS Digital | Does not show |
| The monthly price of the app | £0 | £2.99 |

**Scenario 22 Block 1**

|  | **App 1** | **App 2** |
| --- | --- | --- |
| App description | Short with some details about app features | Long and detailed description of the app and its features |
| The ratings of the app | 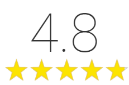 | 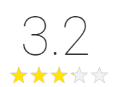 |
| Images shown | Screenshot(s) of the app | Logo and screenshot(s) of the app |
| Who developed the app | NHS Digital | Mhealth Essentials Ltd. |
| The monthly price of the app | £2.99 | £0 |

**Scenario 29 Block 1**

|  | **App 1** | **App 2** |
| --- | --- | --- |
| App description | Generic, to create a rough idea of what the app is about without getting into details of app features | Long and detailed description of the app and its features |
| The ratings of the app | 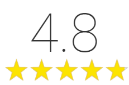 | 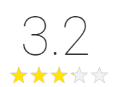 |
| Images shown | Logo of the app | Screenshot(s) of the app |
| Who developed the app | Mhealth Essentials Ltd. | NHS Digital |
| The monthly price of the app | £2.99 | £0 |

**Scenario 32 Block 1**

|  | **App 1** | **App 2** |
| --- | --- | --- |
| App description | Short with some details about app features | Long and detailed description of the app and its features |
| The ratings of the app | 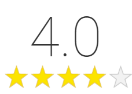 | 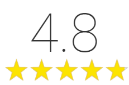 |
| Images shown | Screenshot(s) of the app | Logo of the app |
| Who developed the app | Does not show | Mhealth Essentials Ltd. |
| The monthly price of the app | £2.99 | £8.99 |

**Scenario 33 Block 1**

|  | **App 1** | **App 2** |
| --- | --- | --- |
| App description | Long and detailed description of the app and its features | Generic, to create a rough idea of what the app is about without getting into details of app features |
| The ratings of the app | 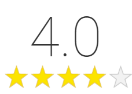 | Does not show |
| Images shown | Logo of the app | Logo and screenshot(s) of the app |
| Who developed the app | NHS Digital | Does not show |
| The monthly price of the app | £5.99 | £0 |

**Scenario 34 Block 1**

|  | **App 1** | **App 2** |
| --- | --- | --- |
| App description | Short with some details about app features | Short with some details about app features |
| The ratings of the app | 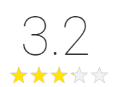 | 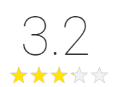 |
| Images shown | Logo of the app | Screenshot(s) of the app |
| Who developed the app | Does not show | Mhealth Essentials Ltd. |
| The monthly price of the app | £8.99 | £8.99 |

**Scenario 38 Block 1**

|  | **App 1** | **App 2** |
| --- | --- | --- |
| App description | Generic, to create a rough idea of what the app is about without getting into details of app features | Short with some details about app features |
| The ratings of the app | 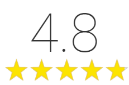 | 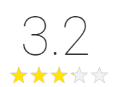 |
| Images shown | Logo and screenshot(s) of the app | Screenshot(s) of the app |
| Who developed the app | NHS Digital | Does not show |
| The monthly price of the app | £5.99 | £0 |

**Scenario 47 Block 1**

|  | **App 1** | **App 2** |
| --- | --- | --- |
| App description | Generic, to create a rough idea of what the app is about without getting into details of app features | Short with some details about app features |
| The ratings of the app | Does not show | 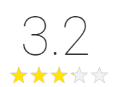 |
| Images shown | Screenshot(s) of the app | Logo and screenshot(s) of the app |
| Who developed the app | Mhealth Essentials Ltd. | NHS Digital |
| The monthly price of the app | £8.99 | £8.99 |

**Block 2**

**Choice situations: 1, 2, 13, 14, 16, 17, 19, 24, 26, 30, 36, 48**

**Scenario 1 Block 2**

|  | **App 1** | **App 2** |
| --- | --- | --- |
| App description | Long and detailed description of the app and its features | Generic, to create a rough idea of what the app is about without getting into details of app features |
| The ratings of the app | Does not show | 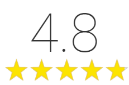 |
| Images shown | Logo of the app | Screenshot(s) of the app |
| Who developed the app | NHS Digital | Mhealth Essentials Ltd. |
| The monthly price of the app | £0 | £2.99 |

**Scenario 2 Block 2**

|  | **App 1** | **App 2** |
| --- | --- | --- |
| App description | Short with some details about app features | Long and detailed description of the app and its features |
| The ratings of the app | 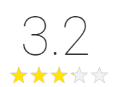 | Does not show |
| Images shown | Screenshot(s) of the app | Logo and screenshot(s) of the app |
| Who developed the app | Mhealth Essentials Ltd. | Does not show |
| The monthly price of the app | £0 | £2.99 |

**Scenario 13 Block 2**

|  | **App 1** | **App 2** |
| --- | --- | --- |
| App description | Short with some details about app features | Long and detailed description of the app and its features |
| The ratings of the app | Does not show | 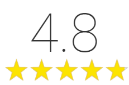 |
| Images shown | Logo and screenshot(s) of the app | Screenshot(s) of the app |
| Who developed the app | Does not show | NHS Digital |
| The monthly price of the app | £0 | £5.99 |

**Scenario 14 Block 2**

|  | **App 1** | **App 2** |
| --- | --- | --- |
| App description | Long and detailed description of the app and its features | Generic, to create a rough idea of what the app is about without getting into details of app features |
| The ratings of the app | 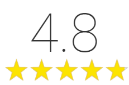 | Does not show |
| Images shown | Logo and screenshot(s) of the app | Logo of the app |
| Who developed the app | Mhealth Essentials Ltd. | NHS Digital |
| The monthly price of the app | £5.99 | £0 |

**Scenario 16 Block 2**

|  | **App 1** | **App 2** |
| --- | --- | --- |
| App description | Short with some details about app features | Generic, to create a rough idea of what the app is about without getting into details of app features |
| The ratings of the app | 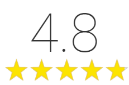 | Does not show |
| Images shown | Logo and screenshot(s) of the app | Logo of the app |
| Who developed the app | Does not show | NHS Digital |
| The monthly price of the app | £0 | £0 |

**Scenario 17 Block 2**

|  | **App 1** | **App 2** |
| --- | --- | --- |
| App description | Generic, to create a rough idea of what the app is about without getting into details of app features | Long and detailed description of the app and its features |
| The ratings of the app | 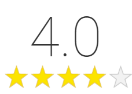 | Does not show |
| Images shown | Logo of the app | Screenshot(s) of the app |
| Who developed the app | NHS Digital | Mhealth Essentials Ltd. |
| The monthly price of the app | £8.99 | £5.99 |

**Scenario 19 Block 2**

|  | **App 1** | **App 2** |
| --- | --- | --- |
| App description | Generic, to create a rough idea of what the app is about without getting into details of app features | Long and detailed description of the app and its features |
| The ratings of the app | 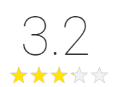 | 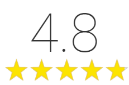 |
| Images shown | Logo and screenshot(s) of the app | Logo of the app |
| Who developed the app | Mhealth Essentials Ltd. | Does not show |
| The monthly price of the app | £0 | £5.99 |

**Scenario 24 Block 2**

|  | **App 1** | **App 2** |
| --- | --- | --- |
| App description | Generic, to create a rough idea of what the app is about without getting into details of app features | Long and detailed description of the app and its features |
| The ratings of the app | 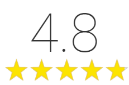 | 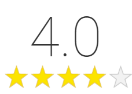 |
| Images shown | Logo and screenshot(s) of the app | Logo of the app |
| Who developed the app | NHS Digital | Mhealth Essentials Ltd. |
| The monthly price of the app | £2.99 | £5.99 |

**Scenario 26 Block 2**

|  | **App 1** | **App 2** |
| --- | --- | --- |
| App description | Generic, to create a rough idea of what the app is about without getting into details of app features | Short with some details about app features |
| The ratings of the app | Does not show | 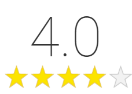 |
| Images shown | Screenshot(s) of the app | Logo of the app |
| Who developed the app | Does not show | NHS Digital |
| The monthly price of the app | £0 | £2.99 |

**Scenario 30 Block 2**

|  | **App 1** | **App 2** |
| --- | --- | --- |
| App description | Short with some details about app features | Generic, to create a rough idea of what the app is about without getting into details of app features |
| The ratings of the app | 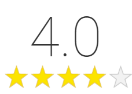 | 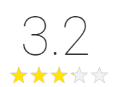 |
| Images shown | Logo and screenshot(s) of the app | Screenshot(s) of the app |
| Who developed the app | NHS Digital | Mhealth Essentials Ltd. |
| The monthly price of the app | £8.99 | £2.99 |

**Scenario 36 Block 2**

|  | **App 1** | **App 2** |
| --- | --- | --- |
| App description | Generic, to create a rough idea of what the app is about without getting into details of app features | Long and detailed description of the app and its features |
| The ratings of the app | 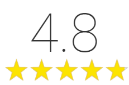 | Does not show |
| Images shown | Screenshot(s) of the app | Logo and screenshot(s) of the app |
| Who developed the app | Does not show | Mhealth Essentials Ltd. |
| The monthly price of the app | £2.99 | £2.99 |

**Scenario 48 Block 2**

|  | **App 1** | **App 2** |
| --- | --- | --- |
| App description | Long and detailed description of the app and its features | Short with some details about app features |
| The ratings of the app | 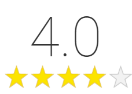 | 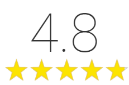 |
| Images shown | Screenshot(s) of the app | Logo of the app |
| Who developed the app | Does not show | Mhealth Essentials Ltd. |
| The monthly price of the app | £8.99 | £8.99 |

**Block 3**

**Choice situations: 3, 7, 11, 12, 20, 25, 28, 31, 39, 43, 44, 45**

**Scenario 3 Block 3**

|  | **App 1** | **App 2** |
| --- | --- | --- |
| App description | Short with some details about app features | Generic, to create a rough idea of what the app is about without getting into details of app features |
| The ratings of the app | 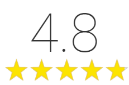 | 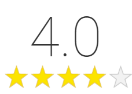 |
| Images shown | Screenshot(s) of the app | Logo of the app |
| Who developed the app | Mhealth Essentials Ltd. | NHS Digital |
| The monthly price of the app | £0 | £2.99 |

**Scenario 7 Block 3**

|  | **App 1** | **App 2** |
| --- | --- | --- |
| App description | Generic, to create a rough idea of what the app is about without getting into details of app features | Long and detailed description of the app and its features |
| The ratings of the app | 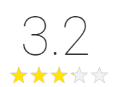 | 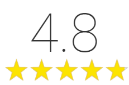 |
| Images shown | Logo and screenshot(s) of the app | Screenshot(s) of the app |
| Who developed the app | NHS Digital | Does not show |
| The monthly price of the app | £0 | £2.99 |

**Scenario 11 Block 3**

|  | **App 1** | **App 2** |
| --- | --- | --- |
| App description | Short with some details about app features | Long and detailed description of the app and its features |
| The ratings of the app | 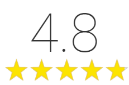 | Does not show |
| Images shown | Logo of the app | Screenshot(s) of the app |
| Who developed the app | Does not show | NHS Digital |
| The monthly price of the app | £2.99 | £2.99 |

**Scenario 12 Block 3**

|  | **App 1** | **App 2** |
| --- | --- | --- |
| App description | Generic, to create a rough idea of what the app is about without getting into details of app features | Short with some details about app features |
| The ratings of the app | Does not show | 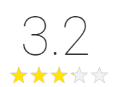 |
| Images shown | Screenshot(s) of the app | Logo and screenshot(s) of the app |
| Who developed the app | Mhealth Essentials Ltd. | Does not show |
| The monthly price of the app | £2.99 | £0 |

**Scenario 20 Block 3**

|  | **App 1** | **App 2** |
| --- | --- | --- |
| App description | Long and detailed description of the app and its features | Short with some details about app features |
| The ratings of the app | Does not show | 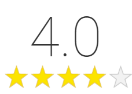 |
| Images shown | Logo and screenshot(s) of the app | Logo of the app |
| Who developed the app | NHS Digital | Mhealth Essentials Ltd. |
| The monthly price of the app | £2.99 | £2.99 |

**Scenario 25 Block 3**

|  | **App 1** | **App 2** |
| --- | --- | --- |
| App description | Generic, to create a rough idea of what the app is about without getting into details of app features | Short with some details about app features |
| The ratings of the app | 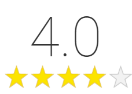 | 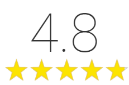 |
| Images shown | Screenshot(s) of the app | Logo of the app |
| Who developed the app | Does not show | NHS Digital |
| The monthly price of the app | £0 | £2.99 |

**Scenario 28 Block 3**

|  | **App 1** | **App 2** |
| --- | --- | --- |
| App description | Short with some details about app features | Generic, to create a rough idea of what the app is about without getting into details of app features |
| The ratings of the app | 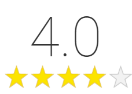 | 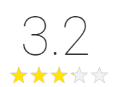 |
| Images shown | Logo and screenshot(s) of the app | Logo of the app |
| Who developed the app | Mhealth Essentials Ltd. | Does not show |
| The monthly price of the app | £8.99 | £5.99 |

**Scenario 31 Block 3**

|  | **App 1** | **App 2** |
| --- | --- | --- |
| App description | Generic, to create a rough idea of what the app is about without getting into details of app features | Short with some details about app features |
| The ratings of the app | 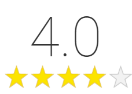 | 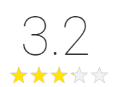 |
| Images shown | Logo of the app | Screenshot(s) of the app |
| Who developed the app | Does not show | NHS Digital |
| The monthly price of the app | £2.99 | £0 |

**Scenario 39 Block 3**

|  | **App 1** | **App 2** |
| --- | --- | --- |
| App description | Short with some details about app features | Long and detailed description of the app and its features |
| The ratings of the app | 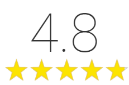 | 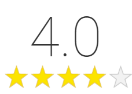 |
| Images shown | Logo of the app | Logo and screenshot(s) of the app |
| Who developed the app | Mhealth Essentials Ltd. | NHS Digital |
| The monthly price of the app | £0 | £0 |

**Scenario 43 Block 3**

|  | **App 1** | **App 2** |
| --- | --- | --- |
| App description | Generic, to create a rough idea of what the app is about without getting into details of app features | Short with some details about app features |
| The ratings of the app | 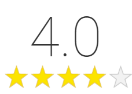 | 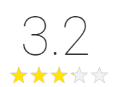 |
| Images shown | Screenshot(s) of the app | Logo of the app |
| Who developed the app | NHS Digital | Mhealth Essentials Ltd. |
| The monthly price of the app | £5.99 | £0 |

**Scenario 44 Block 3**

|  | **App 1** | **App 2** |
| --- | --- | --- |
| App description | Short with some details about app features | Long and detailed description of the app and its features |
| The ratings of the app | 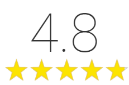 | 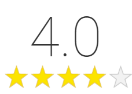 |
| Images shown | Screenshot(s) of the app | Logo and screenshot(s) of the app |
| Who developed the app | NHS Digital | Does not show |
| The monthly price of the app | £8.99 | £8.99 |

**Scenario 45 Block 3**

|  | **App 1** | **App 2** |
| --- | --- | --- |
| App description | Generic, to create a rough idea of what the app is about without getting into details of app features | Long and detailed description of the app and its features |
| The ratings of the app | 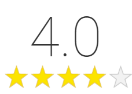 | 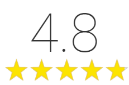 |
| Images shown | Logo of the app | Logo and screenshot(s) of the app |
| Who developed the app | Does not show | NHS Digital |
| The monthly price of the app | £5.99 | £5.99 |

**Block 4**

**Choice situations: 5, 6, 8, 21, 23, 27, 35, 37, 40, 41, 42, 46**

**Scenario 5 Block 4**

|  | **App 1** | **App 2** |
| --- | --- | --- |
| App description | Generic, to create a rough idea of what the app is about without getting into details of app features | Long and detailed description of the app and its features |
| The ratings of the app | 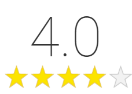 | Does not show |
| Images shown | Logo of the app | Logo and screenshot(s) of the app |
| Who developed the app | Mhealth Essentials Ltd. | NHS Digital |
| The monthly price of the app | £0 | £0 |

**Scenario 6 Block 4**

|  | **App 1** | **App 2** |
| --- | --- | --- |
| App description | Generic, to create a rough idea of what the app is about without getting into details of app features | Short with some details about app features |
| The ratings of the app | 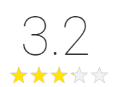 | 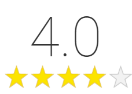 |
| Images shown | Logo of the app | Logo and screenshot(s) of the app |
| Who developed the app | Does not show | Mhealth Essentials Ltd. |
| The monthly price of the app | £2.99 | £5.99 |

**Scenario 8 Block 4**

|  | **App 1** | **App 2** |
| --- | --- | --- |
| App description | Short with some details about app features | Long and detailed description of the app and its features |
| The ratings of the app | 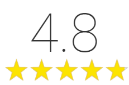 | 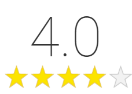 |
| Images shown | Logo and screenshot(s) of the app | Logo of the app |
| Who developed the app | NHS Digital | Mhealth Essentials Ltd. |
| The monthly price of the app | £2.99 | £2.99 |

**Scenario 21 Block 4**

|  | **App 1** | **App 2** |
| --- | --- | --- |
| App description | Short with some details about app features | Long and detailed description of the app and its features |
| The ratings of the app | Does not show | 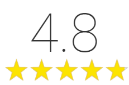 |
| Images shown | Logo of the app | Logo and screenshot(s) of the app |
| Who developed the app | NHS Digital | Mhealth Essentials Ltd. |
| The monthly price of the app | £2.99 | £5.99 |

**Scenario 23 Block 4**

|  | **App 1** | **App 2** |
| --- | --- | --- |
| App description | Long and detailed description of the app and its features | Short with some details about app features |
| The ratings of the app | 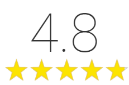 | Does not show |
| Images shown | Screenshot(s) of the app | Logo and screenshot(s) of the app |
| Who developed the app | Mhealth Essentials Ltd. | Does not show |
| The monthly price of the app | £8.99 | £5.99 |

**Scenario 27 Block 4**

|  | **App 1** | **App 2** |
| --- | --- | --- |
| App description | Short with some details about app features | Generic, to create a rough idea of what the app is about without getting into details of app features |
| The ratings of the app | Does not show | 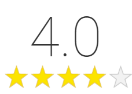 |
| Images shown | Logo of the app | Logo and screenshot(s) of the app |
| Who developed the app | Does not show | NHS Digital |
| The monthly price of the app | £0 | £2.99 |

**Scenario 35 Block 4**

|  | **App 1** | **App 2** |
| --- | --- | --- |
| App description | Long and detailed description of the app and its features | Generic, to create a rough idea of what the app is about without getting into details of app features |
| The ratings of the app | 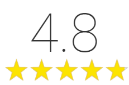 | Does not show |
| Images shown | Logo and screenshot(s) of the app | Screenshot(s) of the app |
| Who developed the app | Does not show | Mhealth Essentials Ltd. |
| The monthly price of the app | £5.99 | £0 |

**Scenario 37 Block 4**

|  | **App 1** | **App 2** |
| --- | --- | --- |
| App description | Generic, to create a rough idea of what the app is about without getting into details of app features | Long and detailed description of the app and its features |
| The ratings of the app | 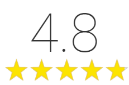 | 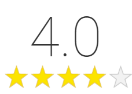 |
| Images shown | Logo of the app | Screenshot(s) of the app |
| Who developed the app | NHS Digital | Does not show |
| The monthly price of the app | £8.99 | £8.99 |

**Scenario 40 Block 4**

|  | **App 1** | **App 2** |
| --- | --- | --- |
| App description | Short with some details about app features | Long and detailed description of the app and its features |
| The ratings of the app | 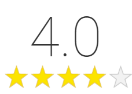 | 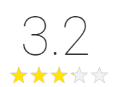 |
| Images shown | Logo and screenshot(s) of the app | Logo of the app |
| Who developed the app | Mhealth Essentials Ltd. | NHS Digital |
| The monthly price of the app | £5.99 | £2.99 |

**Scenario 41 Block 4**

|  | **App 1** | **App 2** |
| --- | --- | --- |
| App description | Generic, to create a rough idea of what the app is about without getting into details of app features | Short with some details about app features |
| The ratings of the app | 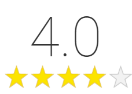 | Does not show |
| Images shown | Logo of the app | Screenshot(s) of the app |
| Who developed the app | Does not show | NHS Digital |
| The monthly price of the app | £2.99 | £0 |

**Scenario 42 Block 4**

|  | **App 1** | **App 2** |
| --- | --- | --- |
| App description | Short with some details about app features | Generic, to create a rough idea of what the app is about without getting into details of app features |
| The ratings of the app | 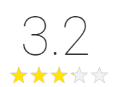 | Does not show |
| Images shown | Screenshot(s) of the app | Logo of the app |
| Who developed the app | Does not show | Mhealth Essentials Ltd. |
| The monthly price of the app | £5.99 | £8.99 |

**Scenario 46 Block 4**

|  | **App 1** | **App 2** |
| --- | --- | --- |
| App description | Generic, to create a rough idea of what the app is about without getting into details of app features | Short with some details about app features |
| The ratings of the app | 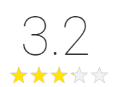 | Does not show |
| Images shown | Logo and screenshot(s) of the app | Screenshot(s) of the app |
| Who developed the app | Mhealth Essentials Ltd. | Does not show |
| The monthly price of the app | £5.99 | £8.99 |

1. **Potential facilitators and barriers of uptake of and engagement with smoking cessation apps and the survey statements to assess these.**

| **TDF Domain** | **Factor** | **U/E/B*** | **Hypothesised facilitator** | **Hypothesised barrier** | **Item in the survey** |
| --- | --- | --- | --- | --- | --- |
| Skills | App literacy | B | Having the ability to use apps confidently. | - | In general, I can easily use a newly installed app on my phone. |
| Knowledge | App awareness | U | - | Lack of awareness of smoking cessation apps. | I was aware of the existence of smoking cessation apps prior to taking part in this study. |
| Knowledge | User guidance | E | Providing knowledge of how to use an app. | - | A guide of how to use features would help me use the app more often. |
| Knowledge | Health information | E | Improves knowledge of own health. | - | Information in the app about how quitting smoking improves my health would make me use the app more often. |
| Memory, attention, decision processes | Cognitive load | E | - | Complicated and time-consuming features. | In general, I don’t want to use an app with features that would take some time to learn. |
| Memory, attention, decision processes | Reminders | E | Help individuals to pay attention on quitting smoking. |  | It would be important that an app to help me quit smoking sends personalised reminders to me. |
| Memory, attention, decision processes | Reminders |  |  | Drawing individuals’ attention on smoking triggering cravings. | I wouldn’t want to use an app that sent me reminders about quitting smoking in case it would trigger my cravings to smoke. |
| Social influence | Peer support | E | Social interaction that promotes quitting. |  | Being connected with other app users would motivate me to stay on track with my intention to stop smoking. |
| Social influence | Peer support |  |  | Social interaction triggers shame or disappointment when one is failing to quit. | Being connected with other app users would make me feel ashamed or disappointed if I started smoking again after quitting. |
| Social influence | Professional support | E | Improves quitting. | - | Being connected with online helpers (quit smoking advisors) within the app would make want to use the app more. |
| Beliefs about capabilities | Self-confidence | E | Promotes quitting smoking by using the app. | - | I am confident I could quit smoking by using an app. |
| Beliefs about consequences | Data protection | B | - | Concern of how the personal data is handled. | I am concerned how my personal data is handled in apps. |
| Goals | Goal setting and action planning | E | - | Goal setting without action planning. | Receiving guidance of how to achieve goals is more important for me than just simply setting goals. |
| Social identity | Social identity | E | - | Using a health app and feeling like a patient. | When using a smoking cessation app, I don’t want to feel that I am being treated like a patient. |
| Reinforcement | Rewards | E | Receiving reward in forms of badges and certificates. | - | Receiving badges or awards for achieving a set goal, would make me use the app more often. |

*U - uptake, E – engagement, B – both uptake and engagement

1. **The questionnaire**

Thank you for your interest in participating in the Health and Wellbeing Smartphone App Research Study. We would like to ask you a few questions to check your eligibility for this study.’*

| **Question** | **Possible answers** | **Eligible if the answer is the following** |
| --- | --- | --- |
| Are you aged 18 or over? | (1) Yes  (2) No | 1 |
| Do you live in the UK? | (1) Yes  (2) No | 1 |
| Do you currently smoke cigarettes? | (1) Yes  (2) No | 1 |
| Do you own or have regular access to a smartphone? | (1) Yes  (2) No | 1 |
| Would you ever consider using a smartphone app to quit smoking cigarettes? | (1) yes  (2) no | 1 |

## **The survey questions**

## **The Discrete Choice Experiment**

Welcome!

In this section of the survey, you will be asked to choose between a few options. The options represent different hypothetical apps to help a smoker quit smoking.

### How to complete this survey

Please consider the following scenario. You wish to quit smoking, and you decide to select a smartphone app to do that. You will need to make a series of choices about which app to select based on the description. In each set of choices, we will present you two options, each of which describes a set of characteristics of smoking apps you might potentially choose. Imagine that these apps are listed on a website that presents information only about health and wellbeing apps as opposed to how these are presented in an app store (e.g. the Apple app store or Google play). The presentation of the apps will describe five characteristics which will be different in each pair. These apps do not actually exist but please answer as if they were real.

Let’s have a look at the characteristics.

1. ***The cost of the app per month*** – this can be any of the following:

- £0
- £2.99
- £5.99
- £8.99

1. ***Who developed the app*** – in some cases you will see the company who developed the app, while in other cases it will not say:

- Doesn’t say
- NHS Digital
- Mhealth Essentials Ltd.

1. ***The user ratings of the app*** – in some cases you will see the ratings of the app, while in other cases it will not say:

- Doesn’t say
- App rated with 3.2 stars
- App rated with 4 stars
- App rated with 4.8 stars

1. ***The app description*** – there are different ways of describing an app, these are the options you will be presented:

- Generic, to create a rough idea of what the app is about without getting into details of app features
- Short with some details about app features
- Long and detailed description of the app and its features

1. ***Images of the app*** – when presenting an app on a website dedicated for health apps can have any of the following picture:

- Logo of the app
- Screenshot(s) of the app
- Logo and screenshot of the app

When you make a choice between the two apps each time, all you need to do is to read the characteristics and choose the option that corresponds to the app you would select. We will remind you about the scenario with each series of choices. Please, take your time when making a decision.

In the next page we will show you a test choice set. Click on the arrow when you are ready to start.

*<Test choice set shown – this will not be included in the data analysis>*

*‘You wish to quit smoking, and you decide to select a smartphone app to do that. Please look at the options carefully, and decide on which app (App 1 or App 2) do you think you would likely want to download and use to help you quit smoking. You could also choose ‘None of these two’ if you do not like either option and would not choose to download either app. Take your time to make a decision.*

*Which app would you choose?*’

<Insert test image>

My answer is:

- App 1
- App 2
- None of these two

Once the choice test is done:

“You will now need to make several choices using the same scenario. Click on the arrow when you are ready to start.”

‘Please, select an option and click on the arrow to continue.’

|  | **App 1** | **App 2** |
| --- | --- | --- |
| The monthly price of the app | £2.99 | £8.99 |
| Who developed the app | NHS Digital | Mhealth Essentials Ltd. |
| The ratings of the app | Does not show | 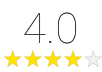 |
| App description | Short with some details about app features | Generic, to create a rough idea of what the app is about without getting into details of app features |
| Images shown | Screenshot(s) of the app | Logo of the app |

My answer is:

- App 1
- App 2
- None of these two

**[If the answer is ‘None of these two’]*

‘We understand that you did not like either option. But imagine that you would have to make a choice. Which one would you prefer?’

My answer is:

- App 1
- App 2

## Uptake and engagement questions

‘Thank you for completing the choice tasks! Now, we would like to know more about your previous experience in using health apps and your views about them. Please, answer the following questions.’

| **Question** | **Your answer to the question is:** |
| --- | --- |
| What type of smartphone do you have or have access to for personal use? | (1) An Android phone  (2) An Apple iPhone  (3) Other type of phone |
| Have you ever used an app designed to help you stop or quit smoking (smoking cessation app)? | (1) Yes  (2) No |
| Have you used any other type of health or wellbeing smartphone app to help you become healthier or to feel better in the last 12 months?  *(For example, apps that can help you drink less alcohol, being more active, losing weight, become less depressed, become less anxious, improve your mood, etc.)* | (1) Yes  (2) No |
| How did you discover the health app(s) you used (i.e. learn about the app’s existence, not where you downloaded it from)? Select all that apply.  [Those who answered yes to ‘Have you ever used a smoking cessation app?’ or ‘Have you ever used another health or wellbeing smartphone app to help you become healthier or to feel better?’ ] | (1) Found via Google search  (2) Found in app store  (3) Found on a health-related website  (2) Recommended by friends or family  (3) Recommended by health practitioners  (4) Other: (free text) |
| When using a health app which of these statements best applies  [Those who answered yes to ‘Have you ever used a smoking cessation app?’ or ‘Have you ever used another health or wellbeing smartphone app to help you become healthier or to feel better?’ ] | (1) I enjoy spending time exploring all the features an app has  (2) I prefer to spend less time on the app, so I would prefer simple features  (3) not sure |

**Please, click the box that most closely corresponds to your feeling regarding each of the statements.**

| **Statements** | **Your answer to the statement is:** |
| --- | --- |
| In general, I can easily use a newly installed app on my phone. | - Strongly agree - Somewhat agree - Neither agree nor disagree - Somewhat disagree - Strongly disagree |
| I was aware of the existence of smoking cessation apps prior to taking part in this study. | - Strongly agree - Somewhat agree - Neither agree nor disagree - Somewhat disagree - Strongly disagree |
| A guide on how to use features will help me use an app more often. | - Strongly agree - Somewhat agree - Neither agree nor disagree - Somewhat disagree - Strongly disagree |
| Information in an app about how quitting smoking improves my health would make me use the app more often. | - Strongly agree - Somewhat agree - Neither agree nor disagree - Somewhat disagree - Strongly disagree |
| In general, I don’t want to use an app with features that would take some time to learn. | - Strongly agree - Somewhat agree - Neither agree nor disagree - Somewhat disagree - Strongly disagree |
| It would be important that an app to help me quit smoking sends personalised reminders to me. | - Strongly agree - Somewhat agree - Neither agree nor disagree - Somewhat disagree - Strongly disagree |
| I wouldn’t want to use an app that sends me reminders about quitting smoking in case it would trigger my cravings to smoke. | - Strongly agree - Somewhat agree - Neither agree nor disagree - Somewhat disagree - Strongly disagree |
| Being connected with other app users would motivate me to stay on track with my intention to stop smoking. | - Strongly agree - Somewhat agree - Neither agree nor disagree - Somewhat disagree - Strongly disagree |
| Being connected with other app users would make me feel ashamed or disappointed if I started smoking again after quitting. | - Strongly agree - Somewhat agree - Neither agree nor disagree - Somewhat disagree - Strongly disagree |
| Being connected with online helpers (e.g. quit smoking advisers) within the app would make me to use the app more. | - Strongly agree - Somewhat agree - Neither agree nor disagree - Somewhat disagree - Strongly disagree |
| I am confident I could quit smoking by using an app. | - Strongly agree - Somewhat agree - Neither agree nor disagree - Somewhat disagree - Strongly disagree |
| I am concerned how my personal data is handled in apps. | - Strongly agree - Somewhat agree - Neither agree nor disagree - Somewhat disagree - Strongly disagree |
| Receiving guidance on how to achieve goals is more important for me than just simply setting goals. | - Strongly agree - Somewhat agree - Neither agree nor disagree - Somewhat disagree - Strongly disagree |
| When using a smoking cessation app, I don’t want to feel that I am being treated like a patient. | - Strongly agree - Somewhat agree - Neither agree nor disagree - Somewhat disagree - Strongly disagree |
| Receiving badges or awards for achieving a set goal would make me use the app more often. | - Strongly agree - Somewhat agree - Neither agree nor disagree - Somewhat disagree - Strongly disagree |

## Smoking and Sociodemographics

‘You are nearly done! We will now ask you a few more questions so we know more about your background. Remember, the information you provide will be anonymised.’

| **Question** | **Your answer to the question is:** |
| --- | --- |
| How many cigarettes per day do you usually smoke? | [free text] |
| How soon do you smoke your first cigarette after you wake-up? | (1) Within 5 minutes  (2) 6 – 30 minutes  (3) 31 – 60 minutes  (4) More than 60 minutes |
| When was the last time you made a serious quit attempt that lasted at least 24 hours? | (1) In the last month  (2) In the last 12 months  (3) Longer than 12 months ago  (4) I haven’t made an attempt to quit smoking before |
| Have you ever used any of the following to help you stop smoking? (Tick all that apply) | (1) Nicotine replacement product (e.g. patches, gum, inhalator)  (2) Zyban (buprorion)  (3) Champix (varenicline)  (4) E-cigarette or vaping device  (5) Attended a stop smoking group  (6) Attended Stop Smoking one-to-one counselling or support services  (7) Phoned a smoking helpline  (8) A book about quitting smoking  (9) Visited a smoking cessation website  (10) Used a smoking cessation app installed on smartphone, tablet or PDA  (11) None of these  (12) Other (free text) |
| How likely are you planning to quit smoking within the next 6 months? | (1) Very unlikely  (2) Unlikely  (3) Maybe, maybe not  (4) Likely  (5) Very likely |
| How determined are you to quit for good? | (1) Not at all  (2) Slightly  (3) Moderately  (4) Very much  (5) Extremely |
| What would be your main reason for quitting smoking? | (1) Health concerns related to COVID-19  (2) Health concerns not related to COVID-19  (3) Pressure or encouragement from others  (4) To save money  (5) To regain control  (6) Other (free text) |

Demographics:

| **Question** | **Your answer to the question is:** |
| --- | --- |
| What year were you born? | (free text) |
| What gender do you identify with? | (1) Female  (2) Male  (3) Non binary/ Gender fluid  (4) Prefer not to say |
| What is your highest educational qualification? | (1) GSCE or equivalent  (2) A levels or equivalent  (3) Degree or equivalent  (4) Postgraduate or equivalent  (5) Other (free text) |
| What was your net (after tax) household income last month? Please include any benefits your household members received. If you are a single person living a shared house or lodging, please, base this on your individual income. | (1) £0 - £999  (2) £1000 - £1499  (3) £1500 - £1999  (4) £2000 - £2499  (5) £2500 - £2999  (6) £3000 - £3499  (7) £3500 - £3999  (8) £4000 - £4499  (9) £4500 - £4999  (10) over £5000  (11) prefer not to say |
| What is your ethnic group? | (1) White  (2) Black  (3) Asian  (4) Arabic  (5) Mixed/multiple ethnic groups  (6) Other ethnic group (free text) |
| What is your sexual orientation? | (1) Heterosexual or straight  (2) Lesbian  (3) Gay man  (4) Bisexual  (5) Queer  (6) Other (free text)  (7) Prefer not to say |
| Do you have any long-standing illness, disability or infirmity? (Long-standing means anything that has troubled you over a period of time or that is likely to affect you over a period of time)? | (1) No  (2) Yes  (3) Prefer not to say |
